# The Genetic Diversity of Enset (*Ensete ventricosum*) Landraces Used in Traditional Medicine Is Similar to the Diversity Found in Non-medicinal Landraces

**DOI:** 10.3389/fpls.2021.756182

**Published:** 2022-01-06

**Authors:** Gizachew Woldesenbet Nuraga, Tileye Feyissa, Kassahun Tesfaye, Manosh Kumar Biswas, Trude Schwarzacher, James S. Borrell, Paul Wilkin, Sebsebe Demissew, Zerihun Tadele, J. S. (Pat) Heslop-Harrison

**Affiliations:** ^1^Department of Genetics and Genome Biology, University of Leicester, Leicester, United Kingdom; ^2^Department of Horticulture, Wolkite University, Wolkite, Ethiopia; ^3^Institute of Biotechnology, Addis Ababa University, Addis Ababa, Ethiopia; ^4^Ethiopian Biotechnology Institute, Addis Ababa, Ethiopia; ^5^Natural Capital and Plant Health Department, Royal Botanic Gardens, Kew, London, United Kingdom; ^6^Department of Plant Biology and Biodiversity Management, Addis Ababa University, Addis Ababa, Ethiopia; ^7^Institute of Plant Sciences, University of Bern, Bern, Switzerland

**Keywords:** conservation, *Ensete ventricosum*, genetic diversity, landrace, SSR markers, traditional medicine

## Abstract

Enset (*Ensete ventricosum*) is a multipurpose crop extensively cultivated in southern and southwestern Ethiopia for human food, animal feed, and fiber. It has immense contributions to the food security and rural livelihoods of 20 million people. Several distinct enset landraces are cultivated for their uses in traditional medicine. These landraces are vulnerable to various human-related activities and environmental constraints. The genetic diversity among the landraces is not verified to plan conservation strategy. Moreover, it is currently unknown whether medicinal landraces are genetically differentiated from other landraces. Here, we characterize the genetic diversity of medicinal enset landraces to support effective conservation and utilization of their diversity. We evaluated the genetic diversity of 51 enset landraces, of which 38 have reported medicinal value. A total of 38 alleles across the 15 simple sequence repeat (SSR) loci and a moderate level of genetic diversity (H_e_ = 0.47) were detected. Analysis of molecular variation (AMOVA) revealed that only 2.4% of the total genetic variation was contributed by variation among the medicinal and non-medicinal groups of landraces, with an F_ST_ of 0.024. A neighbor-joining tree showed four separate clusters with no correlation to the use-values of the landraces. Except for two, all “medicinal” landraces with distinct vernacular names were found to be genetically different, showing that vernacular names are a good indicator of genetic distinctiveness in these specific groups of landraces. The discriminant analysis of the principal components also confirmed the absence of distinct clustering between the two groups. We found that enset landraces were clustered irrespective of their use-value, showing no evidence for genetic differentiation between the enset grown for ‘medicinal’ uses and non-medicinal landraces. This suggests that enset medicinal properties may be restricted to a more limited number of genotypes, might have resulted from the interaction of genotype with the environment or management practice, or partly misreported. The study provides baseline information that promotes further investigations in exploiting the medicinal value of these specific landraces.

## Introduction

Enset (*Ensete ventricosum*; also called Abyssinian banana) is a herbaceous, monocarpic perennial plant that grows from 4 to 10 m in height. It resembles and is closely related to bananas in the genus *Musa*, and these, together with the monotypic genus *Musella*, form the family Musaceae (Borrell et al., 2019). Enset is a regionally important crop, mainly cultivated for starchy human food, animal feed, and fiber. It contributes to the food security and rural livelihoods of a quarter of the population of Ethiopia (Yemataw et al., 2016). It is resilient to extreme environmental conditions, especially to drought (Tsegaye and Struik, 2002) and it is considered a priority crop in areas where the crop is grown as a staple food (Brandt et al., 1997).

The use of indigenous plant species to treat several ailments such as cancer, toothache, and stomach ache in different parts of Ethiopia has been frequently reported (Chekole, 2017; Megersa et al., 2019; Tesfaye et al., 2020). In addition to the extensive use of enset as human food and animal feed, some enset landraces play a well-known and important role in traditional medicine due to their use in repairing broken bones and fractures, assisting the removal of placental remains following birth or an abortion, and for treatment of liver disease (Terefe and Tabogie, 1989; Tsehaye and Kebebew, 2006; Olango et al., 2014). In the comparison of different medicinal plant species, *Ensete ventricosum* was ranked first by the local people for its medicinal use (Tefera and Kim, 2019). A compound that has anti-bacterial and anti-fungal activities extracted from *E. ventricosum* (Hölscher and Schneider, 1998) can be related to the traditional medicinal use of the plant by society. The free amino acid composition analysis of enset landraces indicates that high arginine content could be the other reason for their medicinal properties, as it is associated with collagen formation, tissue repair, and wound healing *via* proline, and it may also stimulate collagen synthesis as a precursor of nitric oxide (Tamrat et al., 2020a). However, experimental studies on different enset landraces claimed to have traditional medicinal importance are scant.

Enset production has been constrained by various plant pests, diseases, and abiotic factors (Merga et al., 2019; Kidane et al., 2021). The loss of some valuable enset genotypes due to various human and environmental factors was also previously reported (Gebremaryam, 1996; Negash et al., 2002). The existence of a gap in collections and conservation of enset landraces was also reported (Dalle and Daba, 2021). Medicinal landraces may be more threatened than others because when a person is ill, the medic is usually given the plant (free of charge) to cure the ailment of the patient, but the farmer does not have an economic reason to propagate and replant the medicinal landraces. Moreover, these landraces are highly preferred by wild animals like porcupines and wild pigs (Negash, 2007) and are more susceptible to diseases and drought (Nuraga et al., 2019a). Since these factors might lead to the complete loss of some of these important landraces, attention needs to be given to the conservation and proper utilization of the landraces that play important roles in traditional medicine.

Conserving domesticated enset diversity as seeds have been considered challenging for several reasons (Tamrat et al., 2020b), and the existing seed conservation measures of the enset crop and its wild relatives is insufficient (Guzzon and Müller, 2016). The most common method of conserving the genetic resources of vegetatively propagated plants like enset is in a field gene bank, which is very costly in terms of requirements for land, maintenance, and labor. In such cases, a clear understanding of the extent of genetic diversity is essential to reduce unnecessary duplication of germplasm (Rao and Hodgkin, 2002). Assessment of diversity using phenotypic traits is relatively straightforward and low cost (Cholastova and Knotova, 2012), and is the first step in identifying duplicates of accessions from phenotypically distinguishable cultivars. However, due to the influence of the environment on the phenotype, evaluating genetic variation at the molecular level is important.

Molecular markers are powerful tools in the assessment of genetic diversity which can assist the management of plant genetic resources (Virk et al., 2000; Teixeira da Silva et al., 2005). Previous enset genetic diversity studies have used molecular techniques including amplified fragment length polymorphism (AFLP; Negash et al., 2002), random amplification of polymorphic DNA (RAPD; Birmeta et al., 2002), inter simple sequence repeat (ISSR; Tobiaw and Bekele, 2011), and simple sequence repeat (SSR; Getachew et al., 2014; Biswas et al., 2020). SSR markers are highly polymorphic, co-dominant and the primer sequences are generally well conserved within and between related species (Karaagac et al., 2014). Recently, (Gerura et al., 2019) and (Olango et al., 2015) have reported the measurement of genetic diversity of enset using SSR markers. The previous studies were carried out on landraces from specific locations, and there was no identification and diversity study on enset landraces used for traditional medicine and other landraces. Therefore, the current study was conducted to investigate the extent of genetic diversity and the relationship that exists within and among enset landraces used in traditional medicine and those having other use-values.

## Materials and Methods

### Plant Material and Genomic DNA Extraction

**FIGURE 1:**
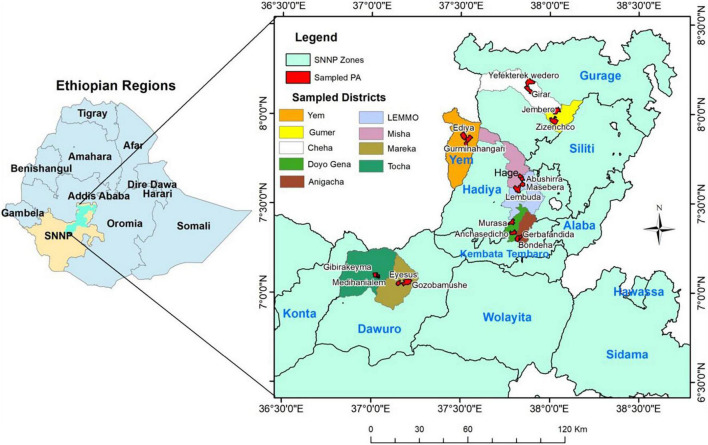
Map of Ethiopia showing its Federal Regions **(left)** and enset sample collection sites that represent the nine studied districts found in, within four zones (Dawuro, Kembata-Tembaro, Hadya, and Gurage), and one special district (Yem) of the Southern, Nations, Nationalities, and Peoples (SNNP) Region. The map was constructed using geographic coordinates and elevation data collected from each sites using global positioning system (GPS). PA, Peasant association (the lowest tier of civil administration unit); SNNP, Southern, Nations, nationalities and Peoples.

Thirty-eight cultivated and named *E. ventricosum* landraces which are used in the treatment of seven different human diseases or disorders were identified with the help of knowledgeable village elders from four locations (administrative zones/special district) consisting of nine districts or special districts of the Southern, Nations, Nationalities, and Peoples (SNNP) regional state of Ethiopia (Figure 1). For comparison, 13 enset landraces that have other non-medicinal use values (principally used for human food) were also sampled. To test the consistency of naming of landraces within each location, up to four duplicate samples (based on their availability) were collected from different sites. Since the landraces are not scientifically characterized, each individual was considered as a separate sample so that a total of 92 plant samples were collected (Supplementary Table 1). The samples were collected from individual farmers’ fields, located at 18 Kebele (the lowest tier of civil administration unit) from across the enset distribution. Since different landraces may have been given the same vernacular name at different locations (Olango et al., 2015), landraces having identical names, but originated from different locations were labeled by including the first letter of names of location after a vernacular name of the second landrace. Healthy young cigar leaf (a recently emerged leaf still rolled as a cylinder) tissue samples were collected from individual plants from November to March 2017 and they were stored in zip-locked plastic bags containing silica gel and preserved until the extraction of genomic DNA. The dried leaf samples were ground and genomic DNA was isolated from 150 mg of each pulverized leaf sample following the modified cetyltrimethylammonium bromide (CTAB) extraction protocol (Borsch et al., 2003).

### PCR Amplification and Electrophoresis

Twenty-one enset SSRs primer-pairs (14 from Olango et al., 2015, and 7 from Biswas et al., 2020) were initially screened for good amplification, polymorphism, and specificity to their target loci using 15 samples. This led to the selection of 15 primer pairs to genotype the landraces (Table 1).

**TABLE 1:** Description and source of the 15 simple sequence repeat (SSR) primers used in genetic diversity of enset landraces.

Marker name	Forward primer sequence (5′–3′)	Reverse primer sequence (5′–3′)	Repeat motif	Size (bp)	References	T_*a*_*^a^* (°C)
Evg-01	AGTCATTGTGCGCAGTTTCC	CGGAGGACTCCATGTGGATGAG	(CTT)_8_	100–120	Olango et al., 2015	60
Evg-02	GGAGAAGCATTTGAAGGTTCTTG	TTCGCATTTATCCCTGGCAC	(AG)_12_	118–153	Olango et al., 2015	62
Evg-04	GCCATCGAGAGCTAAGGGG	GGCAAGGCCGTAAGATCAAC	(AG)_21_	113–147	Olango et al., 2015	60
Evg-05	AGTTGTCACCAATTGCACCG	CCATCCTCCACACATGCC	(GA)_22_	103–141	Olango et al., 2015	62
Evg-06	CCGAAGTGCAACACCAGAG	TCGCTTTGCTCAACATCACC	(GAA)_9_	202–211	Olango et al., 2015	62
Evg-08	CCATCGACGCCTTAACAGAG	TGAACCTCGGGAGTGACATAAG	(GA)_21_	164–190	Olango et al., 2015	60
Evg-09	GCCTTTCGTATGCTTGGTGG	ACGTTGTTGCCGACATTCTG	(GA)_13_	141–175	Olango et al., 2015	60
Evg-10	CAGCCTGTGCAGCTAATCAC	CAGCAGTTGCAGATCGTGTC	(AG)_21_	191–210	Olango et al., 2015	60
Evg-11	GGCCTAGTGACATGATGGTG	TGATGCTAGATTCAAAGTCAAGG	(AC)_13_	135–160	Olango et al., 2015	62
Evg-13	TTGAAAGCATTGCATGTGGC	TCACCACTGTAGACCTCAGC	(CA)_14_	189–229	Olango et al., 2015	62
Evg-14	AACCAATCTGCCTGCATGTG	GCCAGTGATTGTTGAGGTGG	(TGA)_8_	153–159	Olango et al., 2015	62
En^b^	ATCTGCATGCACCCTAGCTT	AAACCCTAACGTCCCTCCTC	(GT)_10_	189	Biswas et al., 2020	62
En^c^	ATCAAGGTCATGTGCTGTGC	ATCAAGGTCATGTGCTGTGC	(CT)_11_	116	Biswas et al., 2020	62
EnM00011571	GATCTGATCCACCTCCTCGT	CGACAAGGATCAAAATGGCT	(AGG)_5_	277	Biswas et al., 2020	64
En^d^	TTCTCTTGCTGCACACACC	TCATGATCCCTGTCCTCCTC	(GA)_9_	313	Biswas et al., 2020	64

*T_a_^a^, annealing temperature; En^b^, EnOnjSSR049028 marker; En^c^, EnBedSSR020585 marker; En^d^, EnM00025665 marker.*

A PCR amplification was carried out in a 20 μl reaction volume containing 1.5 μl (100 mM) template DNA, 11.5 μl molecular reagent water, W 4502 (Sigma, St. Louis, MO, United States), 0.75 μl dNTPs (10 mM) (Bio line, London), 2.5 μl Taq buffer (10× Thermopol reaction buffer), 1.25 μl MgCl_2_ (50 mM), 1 μl forward and reverse primers (10 mM), and 0.5 μl (5 U/μl) BioTaq DNA polymerase (Bioline, London) and amplified using a PCR thermal cycler (BiometraTOne, Terra Universal, Germany). The three-step amplification program consisted of initial (1) denaturation for 2 min at 95°C, (2) 35 cycles of denaturation at 95°C for 1 min, annealing at a temperature specific to each primer set (Table 2), for 1 min, extension at 72°C for 1min, and (3) final extension at 72°C for 10 min. The PCR products were stored at 4°C until electrophoresis.

**TABLE 2:** Levels of diversity indices of the SSR loci.

SSR Loci	N_a_	N_e_	I	H_o_	H_e_	uH_e_	PIC	F
Evg1	3.00	2.27	0.89	0.39	0.54	0.56	0.48	0.30
Evg2	3.00	2.43	0.97	0.42	0.59	0.60	0.52	0.29
Evg4	3.00	2.29	0.92	0.63	0.56	0.57	0.49	–0.12
Evg5	2.00	1.82	0.64	0.54	0.45	0.46	0.36	–0.20
Evg6	2.00	1.41	0.43	0.00	0.27	0.28	0.26	1.00
Evg8	3.00	2.21	0.89	0.51	0.54	0.56	0.50	0.07
Evg9	3.00	2.27	0.93	0.44	0.55	0.56	0.49	0.20
Evg10	2.00	1.82	0.63	0.00	0.44	0.45	0.36	1.00
Evg11	3.00	1.87	0.74	0.41	0.46	0.47	0.43	0.08
Evg13	3.00	2.16	0.85	0.56	0.54	0.55	0.44	–0.06
Evg14	2.00	1.92	0.67	0.64	0.48	0.49	0.37	–0.34
EnO28	3.00	2.70	1.04	0.58	0.63	0.64	0.59	0.08
EnB85	2.00	1.23	0.31	0.21	0.18	0.18	0.16	–0.12
EnM71	2.00	1.91	0.67	0.51	0.48	0.49	0.36	–0.06
EnM65	2.00	1.60	0.56	0.41	0.38	0.39	0.31	–0.09
Mean	2.53	1.99	0.74	0.42	0.47	0.48	0.41	0.14

*N_a_, number of different alleles; N_e_, number of effective alleles; I, Shannon’s information index; H_o_, observed heterozygosity; H_e_, expected heterozygosity; uH_e_, unbiased expected heterozygosity; PIC, polymorphic information content; F, fixation Index; EnO28, EnOnjSSR049028; EnB85, EnBedSSR020585; EnM71, EnM00011571; EnM65, EnM00025665.*

The separation of the amplified product was accomplished in a 4% (w/v) agarose (Bioline, London) gel in 1% (w/v) Tris-acetate-EDTA (TAE) buffer containing ethidium bromide, and electrophoresed at 80 V for 3 h. A standard DNA ladder of 100 bp (Q step 2, Yorkshire Bioscience Ltd., United Kingdom) was loaded together with the samples to estimate molecular weight. The banding pattern was visualized using a gel documentation system (NuGenius, SYNGENE, Cambridge, United Kingdom) and the pictures were documented for scoring.

### Data Scoring and Analysis

The sizes of the clearly amplified fragments were estimated across all the sampled landraces. The number of different alleles (N_*a*_), the effective number of alleles (N_e_), Shannon’s information index (I), observed heterozygosity (H_*o*_), expected heterozygosity (H_e_), un-biased expected heterozygosity (uH_e_), and Fixation index for each locus were computed using GENALEX version 6.503 (Peakall and Smouse, 2012). The Polymorphism Information Content (PIC) for each locus was computed using PowerMarker version 3.25 (Liu and Muse, 2005). The Genetic differentiation (F_ST_) between the two groups of landraces was estimated using GENALEX. An analysis of molecular variation (AMOVA) was performed to evaluate the relative level of genetic variations among groups, and among individuals within a group using GENALEX. The neighbor-joining (NJ) tree was constructed using the software DARwin (Perrier et al., 2003) based on Nei’s genetic distance (Nei, 1972) to reveal the genetic relationships among the groups and individual landraces. The resulting trees were displayed using Fig Tree var.1.4.3 (Andrew, 2016). Discriminant Analysis of Principal Components (DAPC) was implemented using R, version 4.4.1 in ‘adegenet’ package (Jombart, 2008). Detection of admixture was inferred using a Bayesian model-based clustering algorithm implemented in STRUCTURE version 2.3.4 (Pritchard et al., 2000), To determine the most likely number of populations (K), the simulation method of Evanno et al. (2005) was implemented using the web-based STRUCTURE HARVESTER ver. 0.6.92 (Earl and von Holdt, 2012). Each of the probable K was run 10 times with K = 1–10, and the length of burning period was set at 50,000 and 500,000 Markov chain Monte Carlo (MCMC) iterations.

Principal coordinates analysis was carried out using R version 3.6.3 (R Core Team, 2017) to further evaluate the genetic similarity between the landraces.

## Results

Fifteen SSR markers that produced clear and scorable bands were analyzed to evaluate the genetic diversity and the relationship of *E. ventricosum* landraces used in traditional medicine and those having other use-values.

### Genetic Diversity

The polymorphic nature of some of the SSR markers was as shown in Supplementary Figure 1. A total of 38 alleles were detected across 15 SSR loci in 92 genotypes (Table 2). The number of alleles generated per locus ranged from 2 to 3, with an average of 2.53 alleles. The PIC values for the markers varied from 0.16 (primer EnBedSSR020585) to 0.52 (primer Evg2) with an average of 0.41. The observed heterozygosity (H_*o*_) and expected heterozygosity (H_e_) ranged from 0 to 0.64 and 0.18 to 0.63, respectively, and Shannon’s information index (I) ranged from 0.31 to 1.04.

### Genetic Differentiation and Relationships

The AMOVA showed that 97.6% of the total variation was assigned to individuals within a group; while only 2.4% variation was contributed by variation among the groups (Table 3). The overall genetic divergences among the two groups of enset landraces (“medicinal” and “non-medicinal”), measured in coefficients of genetic differentiation (F_ST_) was 0.024 (Table 3).

**TABLE 3:** Analysis of molecular variance and fixation index for landraces used in traditional medicine and those having other use values based on data from 15 loci.

Source of variation	Degree of freedom	Sum of square	Variance components	Percent variation	Fixation index	*P* value
Among groups	1	8.28	0.09	2.4	F_ST_: 0.024	0.008
Within groups	182	669.83	3.68	97.6		
Total	183	678.11	3.77	100		

**FIGURE 2:**
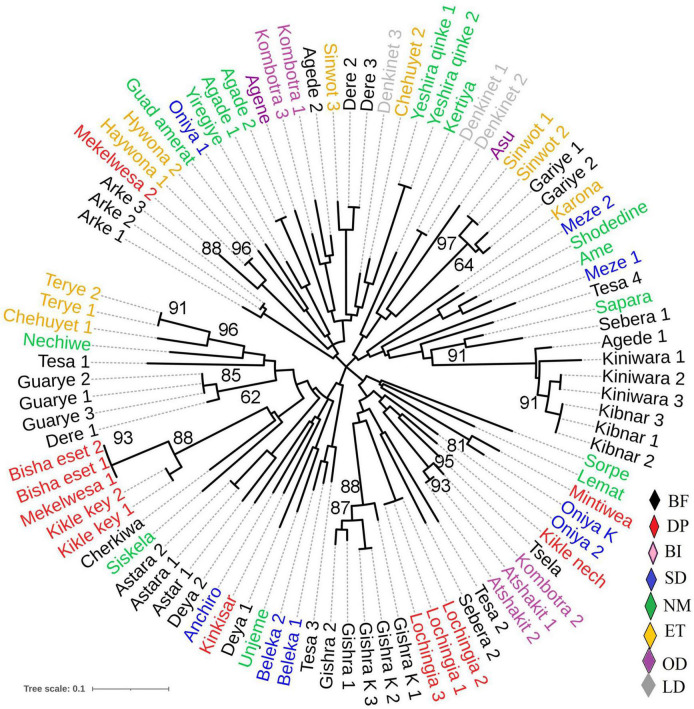
The unrooted neighbor-joining tree generated based on simple matching dissimilarity coefficients over 1,000 replicates, showing the genetic relationship among 51 *Ensete ventricosum* landraces (duplicated on average two times) using 15 SSR markers. Landraces are color-coded according to previously identified diseases types or disorders treated by the landraces traditionally, as designated by: BF for bone fracture; DP, discharge of placenta; BI, back injury; SD, skin itching and diarrhea; NM, non-medicinal; ET, expulsion of thorn and drainage of abscess from tissue; LD, liver disease; OD, other diseases. Numbers at the nodes are bootstrap values only ≥ 60%.

**FIGURE 3:**
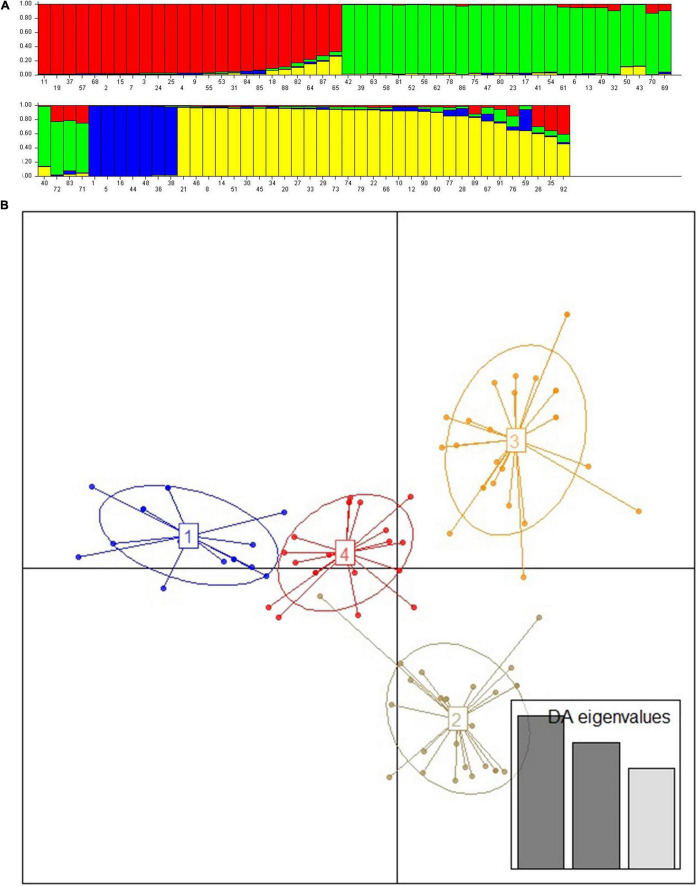
Population structure and detection of admixture based on 15 polymorphic simple sequence repeat (SSR) markers indicating estimated group structure with individual group membership values (1–92 following arrangement of landraces in Supplementary Table 1) **(A)** and Discriminant analyses of principal components (DAPC) scatter plot for 92 enset landraces **(B)**. The axes represent the first two linear discriminants, each circle represents a cluster, and each dot represents an individual. Numbers represent the different subpopulations identified by DAPC analysis.

The unweighted neighbor-joining tree cluster analysis performed using Nei’s genetic distance showed that landraces used in the treatment of a specific disease traditionally were not grouped into the same cluster or sub-cluster; instead, they were mixed with those landraces having other use values (Figure 2). Similarly, the landraces originating from each location were scattered into all 4 clusters (data not presented). In the neighbor-joining tree, it was also observed that some of the landraces with the same vernacular name (replicate samples) were found to be identical, while the others show a difference. Whereas, except two (*bishaeset* and *mekelwesa*), all landraces with the different vernacular names were distinct. The Bayesian clustering result showed the presence of four subpopulations, with some shared admixture memberships (Figure 3A), which is in agreement with the results of the neighbor-joining tree. The DAPC grouped the studied enset landraces into four clusters irrespective of their use value (Figure 3B and Supplementary Table 2). Although the medicinal and non-medicinal landraces were not separately clustered, the majority of the later were grouped in to cluster 3 and 4.

## Discussion

### Genetic Diversity

We assessed the genetic diversity of 38 cultivated enset landraces used in traditional medicine and 13 landraces that have non-medicinal values. According to our results, a moderate level of genetic diversity (H_e_ = 0.47) was detected. A relatively higher H_e_ values (0.55 and 0.59) of enset were reported (Getachew et al., 2014) and (Olango et al., 2015; Gerura et al., 2019), respectively. The value of I (0.74) in the current study was also lower as compared with the earlier report (1.08) (Gerura et al., 2019). The variation of the result is probably due to the fact that our study was focused on a selected group of landraces, those used in traditional medicine. Lower genetic diversity estimates were reported earlier using ISSR (Tobiaw and Bekele, 2011) and RAPD (Birmeta et al., 2002) markers. However, comparisons of detailed diversity estimates from marker systems that have different properties and origins of variation do not allow useful conclusions (Powell et al., 1996; Hamza et al., 2013).

### Genetic Differentiation and Relationships

The genetic differentiation between the landraces used in traditional medicine and those having other use-values was very low (0.024). Genetic differentiation values (0.037) among locations were reported on enset (Gerura et al., 2019), although the direct comparison of different populations is difficult. The AMOVA also showed that the proportion of genetic variation among the two groups of enset landrace was very much limited (2.4%), while the majority was contributed by variation among individuals.

From the landraces that have the same vernacular names (replicate samples), the majority were closely similar genetically and placed together in the neighbor-joining tree. This indicates that farmers have rich indigenous knowledge in identifying and naming enset landraces based on phenotypic traits, and the knowledge is shared across the growing region. However, few other replicates of landraces were placed in different clusters, indicating that genetically different landraces were given the same vernacular name. Perfect identification of genotypes using morphological traits is difficult, and the existence of homonyms has been reported previously (Olango et al., 2015).

Except for two, all landraces with distinct vernacular names were found to be different, showing that vernacular names are good indicators of genetic distinctiveness in these specific groups of landraces. Whereas, the existence of 37 and 8 duplicates of landrace in diversity analysis of enset using four AFLP (Negash et al., 2002) and 12 RAPD (Birmeta et al., 2002) markers, respectively, was reported. Gerura et al. (2019), who studied 83 enset genotypes using 12 SSR markers, also reported 10 duplicates of landraces. Although full identity among the landraces can only be determined when the entire genomes are compared, it is expected that the SSR markers used in the current study could sufficiently discriminate the landraces than the studies that reported a higher number of duplicates. The variation of the results, therefore, could be due to the sample collection method followed in the current study, which involved focusing mainly on specific landraces used in traditional medicine.

The use of some of the enset landraces in traditional treatment of various human ailments in the major enset growing region of Ethiopia, SNNPR, was reported by several authors (Tsehaye and Kebebew, 2006; Olango et al., 2014; Ayenew et al., 2016; Daba and Shigeta, 2016). However, landraces that are used in the treatment of the same types of diseases did not show distinct grouping; instead, landraces used to treat different diseases were mixed with each other and even with those having other use values in the neighbor-joining tree, indicating that “medicinal” properties do not appear to be monophyletic. Furthermore, the DAPC also showed that the two groups of landraces neither formed a separate cluster nor did one group show greater spread or genetic diversity. From these results, it can be argued that landraces that are used in traditional medicine are not genetically distinct from other landraces.

There are several possible explanations for these observations. First, all enset landraces may have a degree of medicinal value, but specific genotypes are preferred for phenotypic or cultural reasons. Second, the medicinal value may arise through genotype-environment interactions or management practices specific to those landraces i.e., they may have non-differentiated genotypes, but *in situ*, they generate unique biochemistry with medicinal properties. Thirdly, a number of important medicinal landraces may have been omitted, or medicinal value incorrectly assigned to non-medicinal landraces. This could serve to hinder our analysis and make it more difficult to detect real genetic differentiation. This would also be an indication of a decline in the quality of indigenous knowledge. We also note that it is unlikely that the strong trust of society upon these landraces could not be developed after a very long period of use, and we have observed remarkable similar enset medicinal claims across a wide variety of distinct ethnic groups in multiple languages. Moreover, anti-bacterial and anti-fungal activities of a compound extracted from the unspecified *E. ventricosum* landrace (Hölscher and Schneider, 1998), and a report (Sreekutty and Mini, 2016) on the medicinal property of a related species, *Ensete superbum*, suggests that at least some of the enset landraces have real medicinal property. The higher mineral concentration (that has a relation with bone health) landraces used in traditional medicine was reported (Nuraga et al., 2019b). Finally, a biochemical survey of enset landraces (Tamrat et al., 2020a) detected high levels of arginine, compared to other amino acids, in three medicinal landraces (*Koshkowashiye*, *Astara*, and *Lochingiya*). Arginine is involved in collagen formation, tissue repair, and wound healing via proline, indicating a possible biochemical basis for the medicinal properties of some of the landraces.

## Conclusion

The study indicated the existence of moderate level genetic diversity among enset landraces used in traditional medicine. The majority of the variation was contributed by variation among individuals, indicating low genetic differentiation among the groups. Except for two, all the landraces with distinct vernacular names were found to be genetically different. The landraces were not clustered based on their use-values, showing no evidence for genetic differentiation between landraces used in traditional medicine and those having other use-values, and the range of diversity in medicinal landraces was little different from that of landraces cultivated for food. In the future, we suggest a biochemical comparison of enset landraces growing in the same environmental and soil condition would complement our analysis, while genetic mapping and genome-wide association studies (GWAS) have the potential to identify genomic regions and genes associated with medicinal traits. The information from this study will be useful for the identification and conservation of enset landraces used in traditional medicine, and it can provide baseline information that promotes further investigations in exploiting the medicinal value of these specific landraces.

## Data Availability Statement

The original contributions presented in the study are included in the article/Supplementary Material, further inquiries can be directed to the corresponding author.

## Author Contributions

GN, TF, KT, and SD designed the experiment in the context of the funded project designed by PW, JH-H, and SD. GN carried out the sample collection, primer design, and laboratory work with MB. GN and MB conducted data mining and carried out the data analysis. GN, JB, TF, and JH-H were major contributors to interpreting the data. All authors contributed background to the design of the work and manuscript writing and revision and approved the final manuscript.

## Conflict of Interest

The authors declare that the research was conducted in the absence of any commercial or financial relationships that could be construed as a potential conflict of interest.

## Publisher’s Note

All claims expressed in this article are solely those of the authors and do not necessarily represent those of their affiliated organizations, or those of the publisher, the editors and the reviewers. Any product that may be evaluated in this article, or claim that may be made by its manufacturer, is not guaranteed or endorsed by the publisher.

## Supplementary Material

Supplementary Figure 1 DNA fragments amplified in selected enset landraces by simple sequence repeat (SSR) primer; a. Evg2 (22 samples) and b. EnM00011571 (16 samples) resolved in agarose gel electrophoresis.
