# Supplementary figures and images for: The Genetic Diversity of Enset (Ensete ventricosum) Landraces Used in Traditional Medicine Is Similar to the Diversity Found in Non-medicinal Landraces
Source: Front Plant Sci. 2022 Jan 6;12:756182. doi: 10.3389/fpls.2021.756182 (PMC8770334; doi:10.3389/fpls.2021.756182)

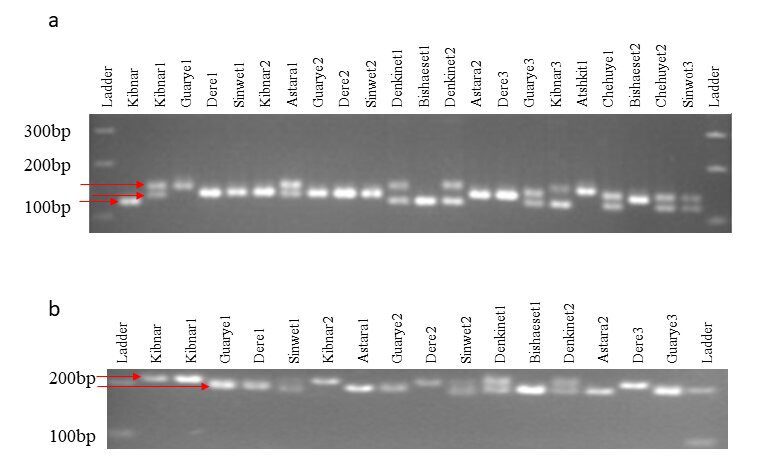

Supplement: Supplementary Figure 1 — DNA fragments amplified in selected enset landraces by simple sequence repeat (SSR) primer; a. Evg2 (22 samples) and b. EnM00011571 (16 samples) resolved in agarose gel electrophoresis. [file Image_1.JPEG]
