# Supplementary material for: The Genetic Diversity of Enset (Ensete ventricosum) Landraces Used in Traditional Medicine Is Similar to the Diversity Found in Non-medicinal Landraces
Source: Front Plant Sci. 2022 Jan 6;12:756182. doi: 10.3389/fpls.2021.756182 (PMC8770334; doi:10.3389/fpls.2021.756182)
Supplement: Supplementary file 2 [file Data_Sheet_1.PDF]

The genotypic and genetic diversity of enset (*Ensete ventricosum*) landraces used in traditional medicine is similar to the diversity found in starchy landraces

Journal of Genetic Resource and Crop Evolution

Gizachew Woldesenbet Nuraga[12]*, Tileye Feyissa, Kassahun Tesfaye, Manosh Kumar Biswas, Trude Schwarzacher, James S. Borrell, Paul Wilkin, Sebsebe Demissew, Zerihun Tadele and J.S. (Pat) Heslop-Harrison

[1]Department of Genetics and Genome Biology, University of Leicester, United Kingdom

[2]Department of Horticulture, Wolkite University, Wolkite, Ethiopia

***Corresponding author** E-mail:bahrangw@gmail.com; Tel: +251 91 334 05 36

Supplementary Table 1 Description of plant materials used in this study; name of enset landraces, major use value and their origin

No.	Local/Vernacular/ names of enset landrace	Major medicinal or other use values of the landrace	Origin of collection		
			Administrative Zone/Special district	District	Kebele/Peasant association/
1	Kibnar1	Treatment of bone fracture	Gurage	Cheha	Yefekterek-Wedro
2	Guarye1	Treatment of bone fracture	Gurage	Cheha	Yefekterek-Wedro
3	Dere1	Treatment of bone fracture	Gurage	Cheha	Yefekterek-Wedro
4	Kibnar2	Treatment of bone fracture	Gurage	Cheha	Yefekterek-Wedro
5	Astara1	Treatment of bone fracture	Gurage	Cheha	Girar
6	Guarye2	Treatment of bone fracture	Gurage	Cheha	Girar
7	Dere2	Treatment of bone fracture	Gurage	Cheha	Girar
8	Astara2	Treatment of bone fracture	Gurage	Gumer	Zizencho
9	Dere3	Treatment of bone fracture	Gurage	Gumer	Zizencho
10	Guarye3	Treatment of bone fracture	Gurage	Gumer	Zizencho
11	Kibnar3	Treatment of bone fracture	Gurage	Gumer	Zizencho
12	Kiniwara1	Treatment of bone fracture	Hadya	Lemo	Lembuda
13	Gishra1	Treatment of bone fracture	Hadya	Lemo	Lembuda
14	Agede1	Treatment of bone fracture	Hadya	Lemo	Lembuda
15	Gishra2	Treatment of bone fracture	Hadya	Lemo	Lembuda
16	Kiniwara2	Treatment of bone fracture	Hadya	Lemo	Lembuda
17	Agede2	Treatment of bone fracture	Hadya	Lemo	Lembuda
18	Kiniwara3	Treatment of bone fracture	Hadya	Misha	Abushira
19	Astar1	Treatment of bone fracture	Hadya	Misha	Abushira
20	Gishra_K1	Treatment of bone fracture	Kembata-Tembaro	Angacha	Gerbafandida
21	Tesa1	Treatment of bone fracture	Kembata-Tembaro	Angacha	Gerbafandida
22	Cherkiwa	Treatment of bone fracture	Kembata-Tembaro	Angacha	Bondena
23	Tesa2	Treatment of bone fracture	Kembata-Tembaro	Angacha	Bondena
24	Gishra_K2	Treatment of bone fracture	Kembata-Tembaro	Angacha	Bondena

25	Sebera1	Treatment of bone fracture	Kembata-Tembaro	Angacha	Bondena
26	Gishra_K3	Treatment of bone fracture	Kembata-Tembaro	Doyogena	Murasa
27	Tesa3	Treatment of bone fracture	Kembata-Tembaro	Doyogena	Anchasedicho
28	Tesa4	Treatment of bone fracture	Kembata-Tembaro	Doyogena	Anchasedicho
29	Sebera2	Treatment of bone fracture	Kembata-Tembaro	Doyogena	Anchasedicho
30	Arke1	Treatment of bone fracture	Dawro	Tocha	Medahnialem
31	Arke2	Treatment of bone fracture	Dawro	Tocha	Medahnialem
32	Arke3	Treatment of bone fracture	Dawro	Tocha	Gibrakeyma
33	Tsela	Treatment of bone fracture	Dawro	Tocha	Gibrakeyma
34	Gariye1	Treatment of bone fracture	Yem[a]	Yem	Gurmina-Hangary
35	Gariye2	Treatment of bone fracture	Yem	Yem	Gurmina-Hangary
36	Deya1	Treatment of bone fracture	Yem	Yem	Gurmina-Hangary
37	Deya2	Treatment of bone fracture	Yem	Yem	Ediya
38	Sinwot1	Expulsion of thorn and drainage abscess from a tissue	Gurage	Cheha	Yefekterek-Wedro
39	Chehuyet1	Expulsion of thorn and drainage abscess from a tissue	Gurage	Gumer	Zizencho
40	Sinwot2	Expulsion of thorn and drainage abscess from a tissue	Gurage	Cheha	Girar
41	Chehuyet2	Expulsion of thorn and drainage abscess from a tissue	Gurage	Gumer	Jemboro
42	Sinwot3	Expulsion of thorn and drainage abscess from a tissue	Gurage	Gumer	Jemboro
43	Terye1	Expulsion of thorn and drainage abscess from a tissue	Gurage	Gumer	Jemboro
44	Terye2	Expulsion of thorn and drainage abscess from a tissue	Gurage	Gumer	Jemboro
45	Hywona1	Expulsion of thorn and drainage abscess from a tissue	Hadya	Lemo	Lembuda
46	Hywona2	Expulsion of thorn and drainage abscess from a tissue	Hadya	Misha	Abushira
47	Karona	Expulsion of thorn and drainage abscess from a tissue	Yem	Yem	Ediya
48	Denkinet1	Treatment of liver disease	Gurage	Cheha	Girar
49	Denkinet2	Treatment of liver disease	Gurage	Cheha	Girar
50	Denkinet3	Treatment of liver disease	Gurage	Gumer	Jemboro

51	Bishaeset1	Discharge of placenta following birth or abortion	Gurage	Cheha	Girar
52	Bishaeset2	Discharge of placenta following birth or abortion	Gurage	Gumer	Zizencho
53	Mekelwesa1	Discharge of placenta following birth or abortion	Hadya	Lemo	Lembuda
54	Mekelwesa2	Discharge of placenta following birth or abortion	Hadya	Lemo	Masbera
55	Kiklenech	Discharge of placenta following birth or abortion	Kembata-Tembaro	Angacha	Gerbafandida
56	Kiklekey1	Discharge of placenta following birth or abortion	Kembata-Tembaro	Angacha	Bondena
57	Mintiwea	Discharge of placenta following birth or abortion	Kembata-Tembaro	Angacha	Bondena
58	Kiklekey2	Discharge of placenta following birth or abortion	Kembata-Tembaro	Doyogena	Anchasedicho
59	Lochingia1	Discharge of placenta following birth or abortion	Dawro	Tocha	Medahnialem
60	Lochingia2	Discharge of placenta following birth or abortion	Dawro	Mareka	Eyesus
61	Lochingia3	Discharge of placenta following birth or abortion	Dawro	Tocha	Gibrakeyma
62	Kinkisir	Discharge of placenta following birth or abortion	Yem	Yem	Gurmina-Hangary
63	Atshakit1	Treatment of back injury	Gurage	Gumer	Jemboro
64	Atshakit2	Treatment of back injury	Gurage	Gumer	Jemboro
65	Kombotra1	Treatment of back injury	Hadya	Lemo	Lembuda
66	Kombotra2	Treatment of back injury	Hadya	Lemo	Lembuda
67	Kombotra3	Treatment of back injury	Hadya	Lemo	Lembuda
68	Oniya1	Treatment of skin itching and diarrhea	Hadya	Lemo	Lembuda
69	Oniya2	Treatment of skin itching and diarrhea	Hadya	Lemo	Lembuda
70	Oniya_K	Treatment of skin itching	Kembata-Tembaro	Angacha	Bondena
71	Beleka1	Treatment of skin itching	Kembata-Tembaro	Doyogena	Anchasedicho
72	Beleka2	Treatment of skin itching	Kembata-Tembaro	Doyogena	Anchasedicho
73	Meze1	Treatment of diarrhea	Dawro	Tocha	Medahnialem
74	Meze2	Treatment of diarrhea	Dawro	Mareka	Gozabamushe
75	Anchiro	Treatment of diarrhea	Yem	Yem	Ediya

76	Agene	Treatment of coughing	Kembata-Tembaro	Angacha	Bondena
77	Asu	For sewing a wound	Yem[a]	Yem	Gurmina-Hangary
78	Guadamerat	Food and other	Gurage	Cheha	Girar
79	Agade1	Food and other	Gurage	Cheha	Girar
80	Sapara	Food and other	Gurage	Cheha	Girar
81	Yiregiye	Food and other	Gurage	Cheha	Girar
82	Yeshiraqinke1	Food and other	Gurage	Cheha	Girar
83	Nechiwe	Food and other	Gurage	Cheha	Girar
84	Lemat	Food and other	Gurage	Cheha	Girar
85	Agade2	Food and other	Gurage	Cheha	Girar
86	Yeshiraqinke2	Food and other	Gurage	Cheha	Girar
87	Unjeme	food and other	Kembata-Tembaro	Doyogena	Murasa
88	Sorpe	food and other	Kembata-Tembaro	Angacha	Bondena
89	Siskela	food and other	Kembata-Tembaro	Angacha	Bondena
90	Shodedine	food and other	Dawro	Mareka	Eyesus
91	Kertiya	food and other	Dawro	Mareka	Eyesus
92	Ame	food and other	Dawro	Mareka	Eyesus

[a] special district
