# Supplementary material for: The Genetic Diversity of Enset (Ensete ventricosum) Landraces Used in Traditional Medicine Is Similar to the Diversity Found in Non-medicinal Landraces
Source: Front Plant Sci. 2022 Jan 6;12:756182. doi: 10.3389/fpls.2021.756182 (PMC8770334; doi:10.3389/fpls.2021.756182)
Supplement: Supplementary file 3 [file Data_Sheet_2.PDF]

The Genetic diversity of enset (*Ensete ventricosum*) landraces used in traditional medicine is similar to the diversity found in Non-medicinal landraces

Journal of Frontiers in Plant Science

Gizachew Woldesenbet Nuraga[12]*, Tileye Feyissa, Kassahun Tesfaye, Manosh Kumar Biswas, Trude Schwarzacher, James S. Borrell, Paul Wilkin, Sebsebe Demissew, Zerihun Tadele and J.S. (Pat) Heslop-Harrison

[1]Department of Genetics and Genome Biology, University of Leicester, United Kingdom

[2]Department of Horticulture, Wolkite University, Wolkite, Ethiopia

***Corresponding author** E-mail:bahrangw@gmail.com; Tel: +251 91 334 05 36

Supplementary Table 2. Enset landraces with their assigned clusters in the discriminant analysis of principal components (DAPC), analyzed using 'Adegenet' package, R software

Names of Landraces	Assigned cluster number	Names of Landraces	Assigned cluster number
Bisha_eset_1	1	Agade_1	3
Bisha_eset_2	1	Agade_2	3
Agede_1	1	Agede_2	3
Dere_1	1	Chehuyet_1	3
Guarye_1	1	Chehuyet_2	3
Guarye_2	1	Gishra_1	3
Guarye_3	1	Gishra_2	3
Kibnar_1	1	Gishra_K_1	3
Kibnar_2	1	Gishra_K_2	3
Kibnar_3	1	Gishra_K_3	3
Kikle_key_1	1	Dere_2	3
Kikle_key_2	1	Dere_3	3
Sapara	1	Denkinet_3	3
Sebera_1	1	Haywona_1	3
Kiniwara_1	1	Haywona_2	3
Kiniwara_2	1	Kombotra_1	3
Kiniwara_3	1	Kombotra_2	3
Mekelwesa_1	1	Kombotra_3	3
Astar_1	2	Mekelwesa_2	3
Kinkisar	2	Oniya_1	3
Kikle_nech	2	Sinwot_3	3

Meze_1	2	Terye_1	3
Siskela	2	Terye_2	3
Mintiwea	2	Yeshira_qinke_1	3
Oniya_2	2	Yeshira_qinke_2	3
Oniya_K	2	Yiregiye	3
Sebera_2	2	Agene	4
Tesa_1	2	Ame	4
Tesa_2	2	Arke_1	4
Tesa_3	2	Arke_2	4
Tsela	2	Arke_3	4
Unjeme	2	Cherkiwa	4
Astara_1	2	Denkinet_1	4
Astara_2	2	Denkinet_2	4
Asu	2	Gariye_1	4
Atshakit_1	2	Gariye_2	4
Atshakit_2	2	Guad_amerat	4
Beleka_1	2	Karona	4
Beleka_2	2	Kertiya	4
Anchiro	2	Meze_2	4
Deya_1	2	Nechiwe	4
Deya_2	2	Shodedine	4
Lemat	2	Sinwot_1	4
Lochingia_1	2	Sinwot_2	4
Lochingia_2	2	Sorpe	4
Lochingia_3	2	Tesa_4	4
